# Unprecedented non-luminal esophageal adenocarcinoma invading the spine

**DOI:** 10.18632/oncoscience.653

**Published:** 2026-03-19

**Authors:** Benjamin Wenyuan Xie, Shreyas Kalantri

**Affiliations:** ^1^University of Louisville School of Medicine, Louisville, KY 40202, USA; ^2^Brown Cancer Center, University of Louisville, Louisville, KY 40202, USA

**Keywords:** esophageal adenocarcinoma, atypical esophageal cancer, spinal invasion, non-luminal presentation, normal esophagogastroduodenoscopy

## Abstract

Background: Upper endoscopy with biopsy is the gold standard for diagnosing esophageal cancer. However, esophageal tumors can rarely escape endoscopic detection by growing in atypical patterns, such as entirely outside the esophageal lumen.

Case: We report a unique case of primary esophageal adenocarcinoma that presented with spinal cord compression due to local invasion of the T5 vertebral body, despite an initially normal upper endoscopy. A 68-year-old man developed progressive paraparesis and sensory loss due to a T5 pathological fracture. He had classic symptoms of esophageal cancer (dysphagia and weight loss), yet endoscopic evaluation one month prior showed no intraluminal tumor. Imaging revealed a posterior mediastinal mass contiguous with the esophagus and invading the T4–T6 vertebrae. Surgical resection of the epidural tumor confirmed poorly differentiated adenocarcinoma consistent with an esophageal primary. The patient declined chemoradiation and succumbed one month later.

Conclusions: This unprecedented pattern of esophageal adenocarcinoma—growing extraluminally with isolated posterior extension to the spine—highlights the potential for esophageal cancer to present with no mucosal lesion on endoscopy. Clinicians should remain vigilant for malignancy in patients with high suspicion, even if initial endoscopic findings are benign.

## INTRODUCTION

Esophageal cancer is a leading cause of cancer-related death worldwide, with two main histologic subtypes: squamous cell carcinoma (SCC) and adenocarcinoma (AC). These subtypes differ in epidemiology and typical tumor location; SCC more often occurs in the upper or mid esophagus, whereas AC usually arises in the distal esophagus (often in the setting of Barrett esophagus) [1]. Despite advances in diagnosis and therapy, esophageal cancer carries a poor prognosis, with an overall five-year survival rate under 20% [2].

Staging of esophageal cancer is based on the Tumor, Node, Metastasis (TNM) staging system (8th edition American Joint Committee on Cancer), wherein T4 disease denotes tumor invasion into adjacent structures [3]. Due to nonspecific early symptoms and the absence of effective screening, many esophageal tumors are diagnosed at advanced stages (T3 or T4) after the tumor has already penetrated the esophageal wall [4]. Tumor invasion in advanced cases commonly involves anterior or lateral structures that neighbor the esophagus such as the tracheobronchial tree, aorta, pleura, or pericardium, since these lie in close contact with the esophageal adventitia. In one analysis of 136 patients with unresectable T4b esophageal SCC, the most frequently involved adjacent organs were the trachea/bronchi (73% of cases) and the aorta/great vessels (45%), whereas direct invasion of the vertebral body was documented in only a single patient [5]. Accordingly, isolated posterior extension of an esophageal tumor into the spine, without any intraluminal or anterior spread, is virtually unprecedented [5, 6]. We present what appears to be the first reported case of an esophageal adenocarcinoma exhibiting such a non-luminal, posterior invasive pattern.

## CASE PRESENTATION

A 68-year-old Caucasian man with no significant past medical history presented with a one-week history of rapidly progressive weakness in both legs, numbness from the mid-chest downward, and new-onset urinary retention. He also reported several months of progressively worsening dysphagia to solid foods, accompanied by unintentional weight loss of approximately 30 pounds over three months. The dysphagia was characterized by a sensation of food sticking in the lower chest and requiring liquids to facilitate passage. He additionally described postprandial substernal discomfort and early satiety, without odynophagia.

On examination, he had diminished sensation below the T6 dermatome, increased deep tendon reflexes with ankle clonus, and near-complete loss of motor function in the lower extremities, findings consistent with thoracic spinal cord compression. Laboratory studies were notable for a hemoglobin of 7.8 g/dL (normocytic anemia); other values, including calcium and alkaline phosphatase, were within normal limits.

**Figure 1:**
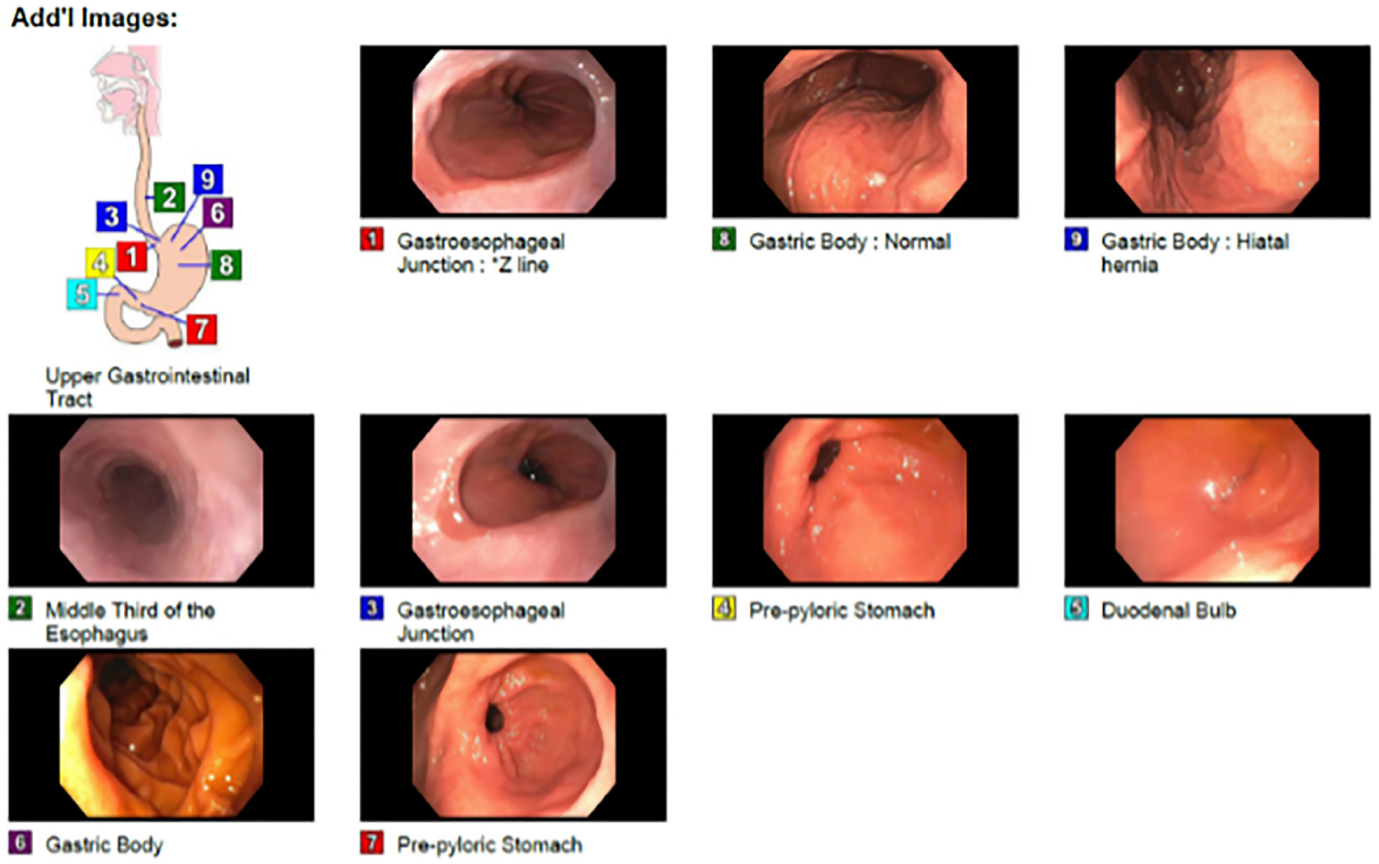
Representative images from diagnostic upper endoscopy performed one month prior to presentation. Images of the gastroesophageal junction (1, 3), middle third of the esophagus (2), and gastric body (6, 8, 9) demonstrate a normal-appearing mucosa without ulceration, mass, or nodularity. Image 9 highlights a small sliding hiatal hernia. Images 4, 7 show a normal pre-pyloric stomach, and image 5 shows an unremarkable duodenal bulb. There were no visible signs of malignancy on endoscopic inspection, underscoring the tumor’s non-luminal growth pattern.

**Figure 2:**
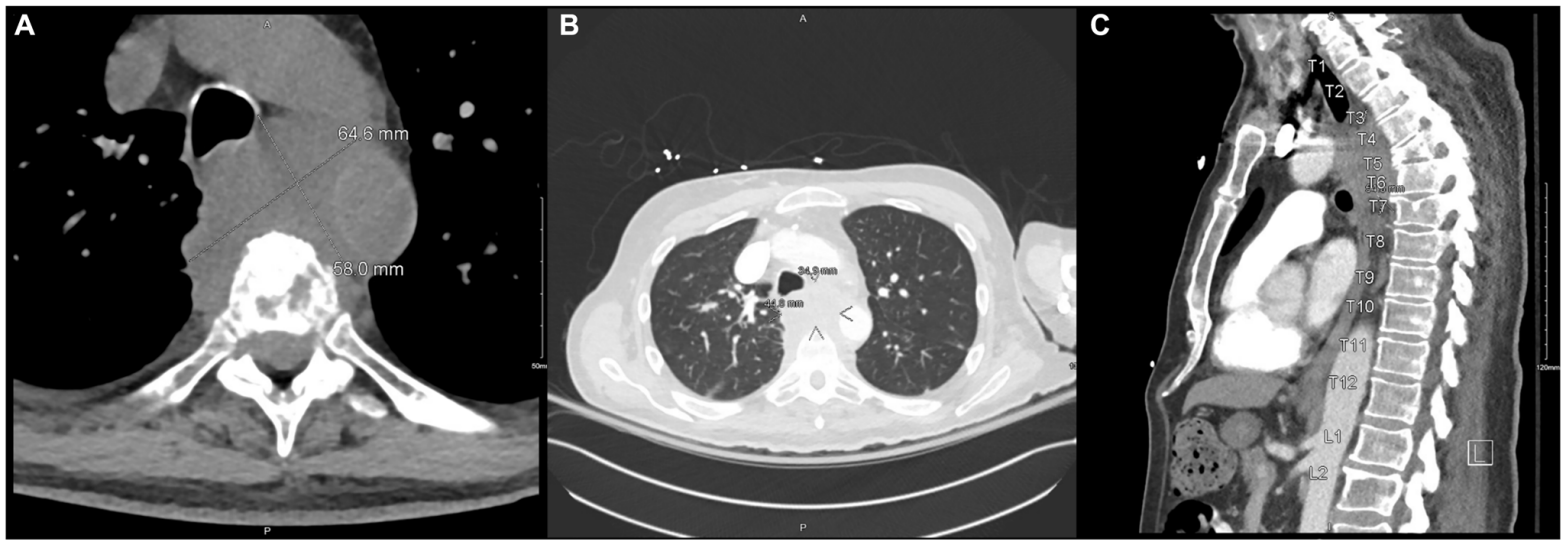
Cross-sectional and sagittal CT images demonstrating the posteriorly invasive esophageal adenocarcinoma. (**A**) Axial CT showing a 64.6 × 58.0 mm posterior mediastinal mass abutting the esophagus and invading the T5 vertebral body. (**B**) Axial lung window showing mediastinal lymphadenopathy and right hilar involvement (34.9 mm and 44.8 mm). (**C**) Sagittal reconstruction revealing extensive T5 vertebral destruction and epidural extension causing spinal canal compromise.

One month prior to admission, the patient had undergone an upper endoscopy for evaluation of his dysphagia and weight loss. That endoscopic exam (with extensive biopsies of the distal esophagus, stomach, and duodenum) revealed only reflux changes and a hiatal hernia; no malignancy or Barrett’s esophagus was identified (Figure 1). Given his new neurological deficits, urgent spinal imaging was obtained. Contrast-enhanced computed tomography (CT) of the chest and spine (Figure 2) demonstrated a pathologic compression fracture of the T5 vertebral body with an associated irregular soft-tissue mass in the posterior mediastinum extending into the paravertebral space. The mass destroyed the T5 vertebra and protruded into the spinal canal, causing significant cord compression. Notably, the lesion was contiguous with the esophagus at that level (no clear fat plane), effectively effacing the posterior esophageal wall; it also abutted the left mainstem bronchus. Multiple pulmonary nodules up to 1.0 cm and right hilar lymphadenopathy were present, suspicious for metastatic disease. CT of the abdomen and pelvis showed ill-defined “misty” nodularity in the mesenteric root (raising concern for peritoneal carcinomatosis, though mesenteric panniculitis was considered) and a few small indeterminate nodules in the right adrenal gland, which were also concerning for metastases.

The patient underwent urgent neurosurgical intervention for spinal cord decompression. A T5 laminectomy with transpedicular resection of the epidural tumor was performed, followed by instrumented stabilization of the spine from T2 to T8. Histopathologic examination of the thoracic spinal lesion demonstrated a poorly differentiated adenocarcinoma with infiltrative growth and marked nuclear atypia. Immunohistochemical staining supported an upper gastrointestinal primary, most consistent with an esophageal origin. Tumor cells were positive for pan-cytokeratin (CK AE1/AE3) and CK7, with patchy cytoplasmic positivity for CK20. CDX-2 demonstrated patchy nuclear immunoreactivity, supporting gastrointestinal differentiation. Markers of pulmonary origin, including TTF-1 and Napsin A, were negative. All immunohistochemical controls reacted appropriately. The diagnosis was metastatic esophageal adenocarcinoma involving the thoracic spine. After multidisciplinary consultation, definitive chemoradiotherapy was recommended due to the tumor’s unresectability; however, the patient declined any further oncologic treatment. Unfortunately, his condition deteriorated, and he died approximately one month after the diagnosis.

## DISCUSSION

We describe an unprecedented case of esophageal adenocarcinoma that evaded detection on initial endoscopy and instead presented with a pathological vertebral fracture caused by localized posterior invasion. Esophageal AC often arises in the setting of Barrett esophagus, with risk factors including chronic gastroesophageal reflux disease (GERD), obesity, and tobacco use [7]. Epidemiologically, the incidence of esophageal AC has been rising rapidly in Western countries, mirroring the increasing prevalence of GERD and obesity [8]. Accordingly, this malignancy now disproportionately affects older Caucasian men in the United States [9]. Our patient fits this profile: he is a 68-year-old white male with long-standing reflux (hiatal hernia) who developed several months of dysphagia and significant weight loss. Despite these risk factors and alarm symptoms, his tumor’s highly atypical growth pattern led to an initially normal endoscopic evaluation, underscoring that even a classic clinical presentation of esophageal cancer can, in rare instances, originate from a lesion that is endoscopically occult.

Several factors may have contributed to the tumor’s atypical behavior in this patient. First, the delay in definitive diagnosis (despite clear warning signs) allowed the cancer to progress unchecked to an advanced stage. The presence of a large hiatal hernia on the initial endoscopy could have provided false reassurance, as it offered an alternate explanation for the patient’s symptoms and may have obscured subtle external compression of the esophagus. It is conceivable that the primary tumor was intramural or otherwise not visible within the lumen during that exam. Second, the anatomical location of the tumor likely facilitated its posterior spread. The esophagus lies immediately anterior to the cervical and upper thoracic vertebral bodies (approximately from C6 through T4) [10]. This intimate anatomical relationship is exemplified by the esophagus often receiving significant collateral dose during high-dose stereotactic radiotherapy of cervicothoracic spine lesions, making it a critical organ at risk in such treatments [11]. In our patient, the malignancy appears to have arisen in the mid-thoracic esophagus near the T5 level, where it had direct access to invade the adjacent vertebral column.

Despite its location, isolated posterior extension of a primary esophageal tumor is highly unusual. Even in tumors arising in the proximal esophagus (a rare location for AC often associated with heterotopic gastric mucosa [12, 13]), invasion into adjacent organs tends to occur anteriorly. Akoum et al. reported a series of 33 cases of primary proximal esophageal AC and noted that in the T4b cases, tumor extension was into anterior structures (trachea or thyroid gland) rather than posteriorly to the spine [12]. Likewise, advanced esophageal SCC more commonly invades the tracheobronchial tree or aorta than the vertebral column [5]. To our knowledge, the presentation of an esophageal adenocarcinoma with exclusive retro-esophageal growth and vertebral destruction—without any intraluminal tumor—has not been previously described. A recent case report (published in abstract form) highlighted an esophageal AC presenting with a pathological spine fracture, but in that case the primary tumor was readily identified as a circumferential distal esophageal mass on endoscopy [14].

Our case is distinct in that the esophageal lesion essentially evaded endoscopic detection entirely. One can only hypothesize that a “perfect storm” of factors led to this scenario: perhaps an atypical tumor origin (e.g., arising from ectopic gastric mucosa in the esophagus) combined with prolonged undisturbed growth allowed the cancer to remain non-luminal until it directly invaded the vertebral body.

From a management standpoint, the standard treatment for a clinically unresectable T4b esophageal cancer (invading major adjacent structures such as the aorta, trachea, or spine) is definitive concurrent chemoradiotherapy rather than surgery [5]. Unfortunately, outcomes in such cases remain poor, with a median overall survival generally less than 10 months even with therapy [15].

One notable consideration in T4b tumors is the risk of esophageal-respiratory fistula formation during chemoradiotherapy. Tumors invading the airway (trachea or bronchi) have a particularly high propensity for developing life-threatening tracheoesophageal fistulas during treatment (incidence around 30%) [5]. By contrast, tumors invading posterior structures like the vertebra are associated with a lower risk of such complications [5]. This could be a small relative advantage in cases like ours, where the tumor’s spread is directed away from the airway.

This case highlights an important diagnostic blind spot in esophageal cancer evaluation, in which reliance on mucosal assessment alone may delay diagnosis in rare extraluminal growth patterns.

Similar cases in the literature provide guidance on appropriate management. In two case series of spinal metastatic esophageal carcinoma (SMEC) involving 20 and 6 patients, prompt neurosurgical intervention was critical in patients with acute neurologic compromise, as timely decompression and stabilization helped preserve overall quality of life [16, 17]. Reported survival following the diagnosis of spinal metastasis ranged from 0 to 14 months, with inflammatory markers such as erythrocyte sedimentation rate, neutrophil-to-lymphocyte ratio, and platelet-to-lymphocyte ratio shown to correlate with overall survival in SMEC patients [16].

Our patient underwent urgent T5 laminectomy with transpedicular resection of the epidural tumor and instrumented stabilization from T2 to T8, a strategy aimed not at oncologic cure but at neurologic salvage and mechanical stabilization. This approach aligns with established principles for the management of malignant spinal cord compression [16, 17].

Together, these findings underscore the need for heightened clinical suspicion of atypical disease presentations, timely surgical palliation in the setting of neurologic compromise, and individualized, goal-directed management strategies for patients with advanced esophageal adenocarcinoma.

## CONCLUSIONS

This case highlights a uniquely unpredictable presentation of esophageal adenocarcinoma. A primary esophageal tumor caused devastating neurologic complications by extending outward to the spine, all while producing no observable lesion within the esophageal lumen on endoscopy. The unusual pattern of spread emphasizes the importance of maintaining a high index of suspicion for malignancy in patients with persistent red-flag symptoms, even if initial diagnostic procedures (such as endoscopy) are unrevealing. Early use of cross-sectional imaging or endoscopic ultrasound should be considered when clinical suspicion remains high, in order to detect atypical tumor spread. Ultimately, this case underscores the unpredictable behavior of esophageal cancer and the need for vigilance in diagnosis. Prompt recognition of such aberrant disease presentations is critical for timely intervention, which may improve quality of life even if prognosis remains poor.

## Abbreviations

AC: adenocarcinoma
CT: computed tomography
GERD: gastroesophageal reflux disease
SCC: squamous cell carcinoma
TNM: Tumor, Node, Metastasis
SMEC: spinal metastatic esophageal carcinoma
